# Methacoline Challenge test as an Evaluator of Response to Statins in Bronchial Hyperresponsiveness

**Published:** 2012

**Authors:** Majid Malek Mohammad, Fanak Fahimi, Atefeh Fakharian, Masoumeh Karimi Gamishan, Mohammad Sistanizad, Nader Fayazi, Soheila Khalilzadeh

**Affiliations:** a *Tracheal Disease Research Center, NRITLD, Masih Daneshvari Hospital , Shahid Beheshti University of Medical Sciences, Tehran, Iran.*; b *Chronic Respiratory Disease Research Center, NRITLD, Masih Daneshvari Hospital , Shahid Beheshti University of Medical Sciences, Tehran, Iran.*; c *Clinical Pharmacy Department, School of Pharmacy, Shahid Beheshti University of Medical Sciences, Tehran, Iran. *; d *Pulmonology Department, TB and Lung Disease Research Center, NRITLD, Masih Daneshvari Hospital.*; e *Internal and Pulmonology Department, Hormozgan University, of Medical Sciences, Iran.*; f *Pediatric Respiratory Disease Research Center, NRITLD, Masih Daneshvari Hospital, Shahid Beheshti University of Medical Sciences, Tehran, Iran. *

**Keywords:** Bronchial hyperresponsiveness, Atorvastatin, Methacholine, Clinical Trial, Lung function, Statin

## Abstract

3-hydroxy-3-methylglutaryl-CoA reductase inhibitors (statins), are effective serum cholesterol-lowering agents which also have anti-inflammatory properties. The objective of this study was to evaluate the effect of atorvastatin on bronchial hyperresponsiveness.

Adult patients (age 14 to 65 years) with bronchial hyperresponsiveness (BHR) diagnosis based on the spirometry with methacholine challenge test were entered into the study. The study was conducted in the National Research Institute of Tuberculosis and Lung Disease. Patients were randomized to receive either atorvastatin 20 mg/day or placebo for 4 weeks. Spirometric parameters were determined at baseline and at completion of the study. Twenty two patients with the age of 32.95±10.30 years completed the trial.

Changes in airway responsiveness categories (moderate to severe, mild, borderline, normal) after the intervention were not significant in atorvastatin group as in placebo group (p-value= 0.131 for atorvastatin group and p-value = 0.305 for placebo group). Also, changes in methacholine solution number (different concentrations of methacholine) which caused at least 20% decrease in FEV1 were not significant between groups (p-value = 0.089). Although we could not find a significant difference, the patients’ fall in FEV1 in atorvastatin group was observed in higher concentrations of methacholine. Median before treatment versus after treatment in atorvastatin group was 1 versus 4 mg/mL, while those were 2 versus 1 mg/mL in placebo group.

This study showed a better but not significant hyperresponsiveness control in the treatment group. The result might be presented more pronounced, if we could increase the sample size.

## Introduction

Statins or 3-hydroxy-3-methylgluteryl coenzyme A (HMG-CoA) reductase inhibitors have pleiotropic anti-inflammatory effects that may be beneficial in the treatment of chronic inflammatory diseases in addition to reducing cholesterol biosynthesis (1, 2). Studies have demonstrated that statins reduce cardiovascular morbidity and mortality in patients with or without coronary artery disease and/or elevated cholesterol levels (3, 4). Administration of atorvastatin can prevent nitrate tolerance in diabetic as well as normal rats (5). Preclinical *in-vitro *and *in-vivo *studies, including experimental models of allergic lung inflammation, have shown that statins decrease components of airway inflammation probably relevant to the pathogenesis of asthma (6, 7). Statins have been shown to reduce the production of interleukin (IL)-6 (8, 9, 10). Statins inhibit T cell activation by decreasing the expression of MHC II on monocytes induced by IFN-*γ *(11). They may also reduce tumor necrosis factor-*α *(TNF-*α*) (12). 

It has been shown that airway hyper- responsiveness is a feature of bronchial asthma, and it has been stressed that airway inflammation has an important role in bronchial hyper- responsiveness in humans (13). Statins have the potential to modify T lymphocyte driven diseases (14-15). 

Th2 lymphocytes are thought to play a key role in the initiation and perpetuation of this airway inflammation, mediated by the functions of their signal cytokines such as IL-3, IL-4, IL-5, and IL-6 (16). There is now evidence that Th1 cells may also contribute to bronchial hyperresponsiveness, and IFN-γ secretion may exacerbate airway inflammation (17-18-19). The potential benefits of statin therapy on inflammatory airway disease were demonstrated in an experimental animal model of allergic airways disease. Simvastatin reduced inflammatory cell infiltrate and eosinophilia in bronchoalveolar lavage (BAL) fluid and decreased IFN-*γ*, IL-4 and IL-5 *in-vitro *(6). The same anti-inflammatory effects of pravastatin have been reported in a similar animal model of allergic airway inflammation (20). Another animal study demonstrated that simvastatin used in emphysema, reduced mRNA expression of IFN-*γ, *TNF-*α *and MMP-12 in the whole lung and reduced the number of neutrophils and lymphocytes and the concentration of TNF-α in BAL fluid, thus reduced inflammation and remodeling (21). The intergroup differences in the anti-inflammatory potency of statins should be kept in mind. It has been suggested that less hydrophil statins such as simvastatin and atorvastatin could have more capacity to suppress inflammation and TNF*-γ *production (22, 23). Though atorvastatin is a stronger inhibitor of the inflammatory response compared to simvastatin, as indicated by NF-kappaB block activation (24). Atorvastatin has been associated with marked down-regulation of HLA-DR and the CD38 activation on peripheral T cells. On the contrary, super antigen-mediated T cell activation was restrained by simvastatin (25). The anti-inflammatory properties of statins are numerous, complex and, although incompletely understood, they so they might prove to be of clinical benefit in the treatment of a range of inflammatory lung diseases. 

Methacholine challenge testing (MCT) is considered when asthma is high possibility and spirometry could not confirm the diagnosis. Methacholine chloride is widely accepted as one of the drugs of choice for bronchial challenge testing to assess airway hyperresponsiveness in both clinical and research settings (26). 

The objective of this study was to evaluate the effect of atorvastatin on FEV1 and bronchial hyperresponsiveness using MCT.

## Experimental

*Study design*

This study was a randomized, double-blinded, placebo-controlled clinical trial with 2 treatment groups comparing the effect of oral atorvastatin 20 mg (Sobhan Pharmaceuticals, Iran) daily for 1 month with that of a matched placebo on the lung function. The study protocol was registered, reviewed and approved by Australian New Zealand Clinical Trial Registry and the registry number is ACTRN12609000704291. 

**Figure 1:**
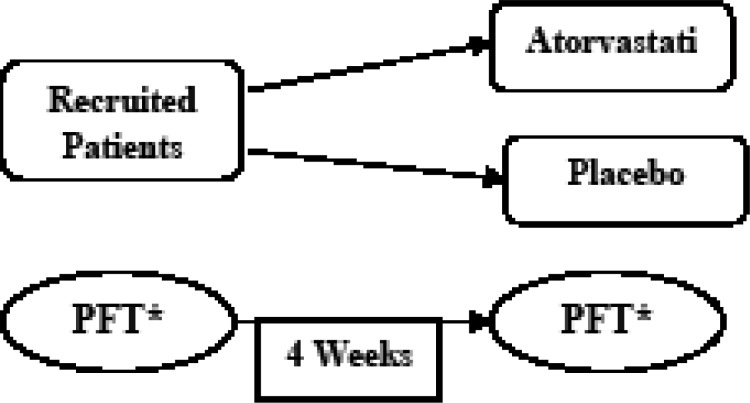
Illustration of the study design *PFT: Pulmonary Function Test

The study was conducted in the pulmonary clinic of the National Research Institute of Tuberculosis and Lung Disease (NRITLD). Consent form was obtained from all the patients entered the study. Patients were randomly allocated to receive either medicine or placebo in a double-blind fashionl (Figure 1). 

All investigators (data collecting investigator, outcome assessor, clinical trial consultant and study statistician) were masked to the treatment allocation of the patients. 

Methacholine preparation

Methacholine powder (Sigma-Aldrich, Fluka, US) has been used and the following 8 doubling concentrations of methacholine in sterile vials were prepared. 

Solution number:

1                2     3      4     5      6      7      8

Concentration (mg/mL):

0.125      0.25      0.5      1      2      4      8      16

*Patients*

Adult patients (age 14 to 65 years) with Bronchial Hyperresponsiveness (BHR) diagnosis based on the spirometry with methacholine challenge test and clinical symptoms were entered into the study. The following exclusion criteria were applied: cardiovascular disease, hepatic disease, acute upper and lower airway infections, use of any lipid lowering drugs during the past six months, use of concurrent drugs affecting atorvastatin metabolism, pregnancy or lactation, clinically significant rise in creatinine phosphokinase (CPK), alanine aminotransferase (ALT) and aspartate aminotransferase (AST) levels, appearance of severe statin side effects e.g. myopathy), secondary diseases that cause worsening of BHR symptoms, using cholinesterase inhibitor agents, uncontrolled hypertension and aortic aneurysm during the study. 

All the laboratory tests were confirmed by the pathologist. After confirmation of normal lipid profile, participants were randomized to receive either placebo or atorvastatin orally 20 mg once daily, for 4 weeks. Allocation concealed by sealed opaque envelopes. The pharmacist dispensed either active drug or placebo according to randomization table from a statistic book. 

*Spirometry and MCT*

Study outcomes were spirometric parameters *i.e. *peak expiratory flow rate (PEFR) and forced expiratory volume in the 1^st^ second (FEV1). After baseline spirometry the concentration was sequentially increased one concentration step at a time, until a decrease in FEV1 greater than 20 percent was seen. The lowest concentration of methacholine which caused ≥ 20% (PC_20_) fall in FEV1 was considered as BHR, while the lack of a 20% fall in FEV1 at the highest concentration (16 mg/mL) ruled out BHR (27). These parameters were measured at baseline and 4 weeks post drug or placebo administration. 

Airway responsiveness was categorized as: PC_20_ <1 mg/mL = moderate to severe BHR, 1- 4 mg/mL = mild BHR, 4 – 16 mg/mL = borderline, >16 mg/mL = normal based on ATS guideline (28). 

*Data analysis*

Data were analyzed using the Statistical Package for Social Sciences (SPSS 16.0, SPSS Inc., Chicago, IL, USA). Independent and paired sample t-test, Mann-whitney and Wilcoxon signed ranks tests were used for comparisons. The results are expressed as mean ± SD, median, ranges. p < 0.05 was regarded as significance level.

## Results

Twenty two patients with BHR diagnosis (12 females and 10 males) with mean±SD age of 32.95 ± 10.30 years could complete the entire 4 weeks fully blinded study protocol. 

The number of patients who received atorvastatin 20 mg/day versus those received placebo was 9 versus 13. The minimum duration of BHR symptoms in patients before the start of the study was one month with mean ± SD duration of 16.60 ± 84.53 months. The chief complaints of these patients were cough (22.7%), dyspnea (18.2%), dry cough (4.5%), and palpitation (4.5%). 36.4% of them had a history of allergic rhinitis. Among all, 27.3% had a family history of asthma and 18.2% had a family history of allergic rhinitis. Drug sensitivity in 9.1% and food allergy in 27.3% of the patients were reported. 

The measured parameters, before and after atorvastatin and placebo therapy are shown in Table 1.

**Table 1:** Spirometric parameters of subjects before and after treatment

Patient	Before treatment				After treatment			group	change in methacholine solution*	
	FEV1 (%)	PC20 (mg/ml)	AR category	FEV1 (%)		PC20 (mg/ml)	AR category		
1	98	4	borderline	93		1	mild	Placebo	-2
2	90	8	borderline	90		16	normal	Placebo	1
3	92	0.25	moderate to severe	88		0.125	moderate to severe	Drug	-1
4	112	8	borderline	109		2	mild	Drug	-2
5	103	4	borderline	104		4	borderline	Placebo	0
6	76	1	mild	77		1	mild	Drug	0
7	103	0.5	moderate to severe	98		16	normal	Drug	5
8	84	4	borderline	80		0.5	moderate to severe	Placebo	-3
9	78	4	borderline	78		2	mild	Placebo	-1
10	110	4	borderline	110		4	mild	Placebo	0
11	88	2	mild	89		0.25	moderate to severe	Placebo	-3
12	109	1	mild	88		2	mild	Placebo	1
13	90	2	mild	91		4	borderline	Drug	1
14	93	1	mild	94		1	mild	Placebo	0
15	116	1	mild	119		1	mild	Placebo	0
16	78	0.25	moderate to severe	86		1	mild	Drug	2
17	81	1	mild	93		16	normal	Drug	4
18	102	4	borderline	100		8	borderline	Drug	2
19	109	8	borderline	103		8	borderline	Drug	0
20	117	0.5	moderate to severe	110		0.5	moderate to severe	Placebo	0
21	90	0.5	moderate to severe	90		8	borderline	Placebo	4
22	82	2	mild	90		0.25	moderate to severe	Placebo	-3

AR: Airway responsiveness* Increase or decrease in solution concentration which caused more than 20% decrease in FEV1

Data analysis revealed no significant differences between baseline FEV1% in the two groups (t= 0.539, p-value= 0.596). Changes in airway responsiveness categories (*e.g*. from mild to borderline airway responsiveness) were not significant in atorvastatin group and placebo group (p-value= 0.131 for atorvastatin group and p value = 0.305 for placebo group). Changes in methacholine solution number which caused greater than 20% decrease in FEV1 were not significant between groups, either (p-value = 0.089). Descriptive parameters of spirometric findings in placebo and atorvastatin group are shown in Table 2. 

There were no significant difference between the alterations of PEFR, FEV1 and FEV1% in neither placebo nor atorvastatin group.

**Table 2:** Descriptive parameters of spirometeric findings in placebo and atorvastatin group

		**Median**	**Minimum**	**Maximum**	**Percentile 25**	**Percentile 75**
**Placebo**	**PC20 before (mg/mL)**	2	0.5	8	1	4
	**PC20 after (mg/mL)**	1	0.25	16	0.5	4
	**Airway responsiveness category (before treatment)**	2	1	3	2	3
	**Airway responsiveness category (after treatment)**	2	1	4	1	2
	change in methacholine solution* (solution number)	0	-3	4	-2	0
**Drug**	**PC20 before (mg/mL)**	1	0.25	8	0.5	4
	**PC20 after (mg/mL)**	4	0.125	16	1	8
	**Airway responsiveness category (before treatment)**	2	1	3	1	3
	**Airway responsiveness category (after treatment)**	3	1	4	2	3
	change in methacholine solution* (solution number)	1	-2	5	0	2

* Increase or decrease in solution number which caused more than 20% decrease in FEV1

## Discussion

According to the results of this study, using atorvastatin 20 mg/day for a month had no significant effect on spirometric indices of BHR normolipid patients. Although we could not find a significant difference, there was a trend in treatment group *i.e. *changes in methacholine solution number, which shows that the patients in that group had drop in FEV1 in higher concentrations of methacholine. 

Median of PC_20_ in atorvastatin group before treatment was less than after treatment (1 versus 4 mg/mL), while those were 2 versus 1 mg/mL in placebo group. This could be considered a better hyperresponsiveness control in the treatment group. This is the first study on BHR patients with statins. The result may be presented more pronounced, if we could increase the sample size. Although many studies have been done in this area but due to complexity and the nature of inflammatory disease, there are controversies about the anti-inflammatory effects of statins and their effect on the inflammatory disease. The result of this study is in contravention with some of the findings of previous studies that have been successful to report anti-inflammatory effects of statins (2, 6, 29, 30, 31).

Of course there are contradictions in the statins effects reported in the previous studies (22, 32, 33, 34).The effects of statins have mainly been studied on cellular markers and few clinical trials on human have been done (14, 20, 32-34).To our knowledge the effects of atorvastatin on human asthma and BHR are unexplored clearly. However, in 2004 Mckay and colleagues examined the anti-inflammatory activity of simvastatin in a murine model of allergic asthma. The result has shown a reduction in IL-4, IL-5, IL-6, and IFN-*γ *secretion in thoracic lymph node cultures from simvastatin-treated mice. This study was conducted on allergic asthma, while we studied on patients with Bronchial Hyperresponsiveness (BHR). In animal trial study, there were two ways of administration: Intra peritoneal (IP) and oral administration. One study has shown IP that route obtained better results (6).This is probably due to first-pass hepatic metabolism of the drug after absorption from the gastrointestinal tract. In 2006 Verhoeven evaluated atherosclerotic plaques in 378 patients that more than half of them were taking different doses of statins. Although IL-6 levels in the group who were taking statins had a decrease, but IL-8 levels in the two groups did not differ and CD68 as a marker of macrophages in the atorvastatin group had increased. While that of IL-4, IL-5, IL-6 as inflammatory mediators involved in BHR (35). In 2006, thirty young healthy male participants who received an injection of the bacterial cell wall endotoxin product to induce systemic inflammation were administered simvastatin 20 mg daily for 14 days. Plasma cytokines (TNF-alpha, IL-6 and IL-1) as well as total leukocyte counts increased in all participants upon endotoxin challenge but were not affected by simvastatin treatment unlike study done by Erikstrup *et al*. (36). 

Evidence of anti-inflammatory effects of statins in autoimmune disease is also obtained. Lawman and colleagues in 2004 showed reduced progression of lupus in mice by atorvastatin (37). It should be noted that lupus is a complex inflammatory disease with a systemic inflammatory while asthma is a localized inflammation and TH2 dominant disease. Further studies were carried on to evaluate the effect of statins in autoimmune diseases with TH1 dominant such as rheumatoid arthritis. Also, there is no evidence about the effect of statins on inflammation induced by Th2-interference (30).

In a similar study in 2008, 44 patients with allergic asthma were treated with atorvastatin 40 mg and placebo for 8 weeks. After 6 weeks of washout, a second 8-week treatment period was carried on. Spirometric parameters, inflammatory markers such as IL6, IL5, IL8, TNF*α*, B4 leukotriene, CRP, NO levels in exhaled breath and blood samples at the beginning, middle and end of each period was measured. The results showed no improvement and no decrease in spirometric indices and inflammatory parameters in these patients (38). The study duration was longer than our study (4-6 weeks). 

CRP levels decreased in previous studies after taking atorvastatin over 4 weeks and in another study after only 2 days (39, 40). Also the anti-inflammatory effects of simvastatin in animal models with allergic asthma have been observed after 28 days (6). In contrast, in another study that was conducted in 2007 by Menzies, there was no anti-inflammatory effect after taking simvastatin for 1 month in asthmatic patients (22). Based on these findings, it is clear that duration of using statins does not seem to determine anti-inflammatory effects of them and 4 weeks should be long enough for study the anti-inflammatory effects of statins. In the study which was conducted in the University of Glasgow, all patients have been used inhaled corticosteroids with atorvastatin simultaneously. Although, this did not change spirometric indices significantly, it is possible that reduction in amount of macrophages and leukotrienes B4 in the sputum of patients was due to synergistic effect of inhaled corticosteroids with atorvastatin (41). We did not determine macrophages and other inflammatory markers in sputum of patients. 

Besides, we used atorvastatin 20 mg/day which failed to make a difference in the outcomes. 

Kiener and colleagues also showed that lipophylic statins such as simvastatin has more anti-inflammatory effect in human and mouse models than pravastatin (a hydrophilic statin) (23). In contrast Joukhadar and colleagues by comparing atorvastatin, simvastatin and pravastatin have shown no difference between their effect on inflammatory parameters (5). There is a possibility that using other statins may increases anti-inflammatory effects in comparison with atorvastatin, which according to some studies this possibility is not far-fetched (6, 20). 

In the study conducted by Menzies and colleagues before starting treatment, using inhaled corticosteroids were prohibited and patients only could use inhaled long-acting beta-agonists (22). According to the reports of other study anti-inflammatory effects of other drug classes can cover the inflammatory effects of statins (42). 

In a new study, 40 asthma patients were studied for one year. Twenty patients received statin and the rest were not taking statins. Patients taking statins showed more decrease in lung function compared to controls (43). 

In conclusion, administration of oral atorvastatin 20 mg/day for 4 weeks did not illustrate a significant impact on spirometric parameters and airway responsiveness in normolipidemic BHR patients.

Since we had only assessed the effects on clinical symptoms and spirometric parameters, we could not conclude that statins lack anti-inflammatory properties at tissue, endothelial or cellular levels. Although the response to different concentrations of methacholine did not show a significant difference between the treatment and placebo group of patients, a trend was observed. Interestingly, patients in atorvastatin group were provoked in higher concentrations while this was reversed in placebo group.

Methacholine challenge test might be a better predictor of the probable effect of statins in BHR patients. Though, for more accurate conclusion on the anti-inflammatory effect of HMG CoA reductase inhibitors, study of the systemic inflammatory biomarkers and inflammatory changes at tissue, endothelial or cellular levels of the respiratory system with a larger sample size is recommended.
